# Recessive Dystrophic *Epidermolysis bullosa* due to Hemizygous 40 kb Deletion of *COL7A1* and the Proximate *PFKFB4* Gene Focusing on the Mutation c.425A>G Mimicking Homozygous Status

**DOI:** 10.3390/diagnostics12102460

**Published:** 2022-10-11

**Authors:** Alfred Klausegger, Niklas Jeschko, Markus Grammer, Jan Cemper-Kiesslich, Franz Neuhuber, Anja Diem, Hannelore Breitenbach-Koller, Gabriele Sander, Dieter Kotzot, Johann Wolfgang Bauer, Martin Laimer

**Affiliations:** 1 EB House Austria, Department of Dermatology and Allergology, University Hospital of the Paracelsus Medical University, 5020 Salzburg, Austria; 2 Department of Legal Medicine, University of Salzburg, 5020 Salzburg, Austria; 3 Department of Biosciences, University of Salzburg, 5020 Salzburg, Austria; 4 Institute of Human Genetics, Department of Pediatrics, University Hospital of the Paracelsus Medical University Salzburg, 5020 Salzburg, Austria; 5 Department of Dermatology and Allergology, University Hospital of the Paracelsus Medical University, 5020 Salzburg, Austria

**Keywords:** *Epidermolysis bullosa*, RDEB, deletion, *COL7A1*, hemizygosity, MLPA, LAM-PCR

## Abstract

Background: Dystrophic *Epidermolysis bullosa* (DEB) is a rare inherited mechanobullous disease characterised by the hyperfragility of the skin and mucous membranes. It is (typically) caused by (loss-of-function) mutations in the *COL7A1* gene that impair the formation of collagen type VII, which represents the major constituent of anchoring fibrils within the basement membrane zone of epithelialised tissues. In a 4-year-old patient diagnosed with the clinical features of recessive DEB, genotyping via Next-Generation EB Panel Sequencing initially revealed the homozygosity of the maternal c.425A>G mutation, while the paternal heterozygosity in exon 3 was lacking. This genetic profile suggested incongruent gene transmission due to uniparental isodisomy (UPD) or the occurrence of a hemizygous deletion of unknown size. Methods: Thus, the EB panel sequencing of genomic DNA, followed by a paternity test and analysis of microsatellite markers, as well as multiplex ligation-dependent probe amplification (MLPA) copy number analysis using patient and parental DNA, were performed. Results: This approach revealed a paternally derived hemizygous deletion spanning from exon 3 to exon 118. Linear amplification-mediated PCR (LAM-PCR) determined the breaking points within intron 2 of the COL7A1 gene, comprising a 40kb segment within intron 1 of the adjacent *PFKFB4* gene. Conclusion: This report highlights the relevance of advanced molecular profiling to determine new/exceptional/unusual genotypes and the accurate mode of genetic transmission in DEB.

## 1. Introduction

Dystrophic *Epidermolysis bullosa* (DEB) is a rare genodermatosis characterised by the hyperfragility of epithelialised tissues with mechanically induced blistering of the skin and mucous membranes. DEB shows either a dominant (DDEB; MIM# 131750) or recessive (RDEB; MIM# 226600) trait of inheritance. Both subtypes result from mutations in the *COL7A1* gene located on chromosome 3p21.3 that encodes for collagen type VII [1]. This protein is the major component of anchoring fibrils that mediate dermal-epidermal anchorage within the basement membrane zone. *COL7A1* spans 31kb on chromosome 3p21.31, and its 8.8 kb coding sequence is segmented into 118 exons [2]. *COL7A1* mutations thus impair the formation and function of anchoring fibrils with subsequent blistering beneath the lamina densa [3].

While DDEB arises from dominant negative glycine mutations, RDEB is mainly caused by compound heterozygous missense (intermediate RDEB) or homozygous loss-of-function (severe RDEB) mutations [4,5]. More than 1000 *COL7A1* mutations have hitherto been reported (http://www.hgmd.cf.ac.uk/ac/gene.php?gene=COL7A1) (accessed on 1 July 2022), including missense, nonsense, splicing, and indel mutations. This genotypic diversity also accounts for the considerable phenotypic variability in DEB [6,7,8,9]. 

Incongruent gene transmission is an extremely rare mode of inheritance. It may result from uniparental disomy (UPD), in which only one parental part of the chromosome is inherited, leading to either paternal or maternal homozygosity [10]. Uniparental isodisomies have been described in a number of genodermatoses, including Netherton syndrome [11,12]. In EB, a total of nine patients with UPD have hitherto been reported, [12,13,14,15,16,17,18,19] and thereof two DEB cases with *COL7A1* mutations [20,21]. Another additional cause of destabilised gene transmission includes extensive microdeletions that have occurred in four DEB patients, involving segments of 851 bp to 520 kb in size [22,23,24,25].

**Figure 1:**
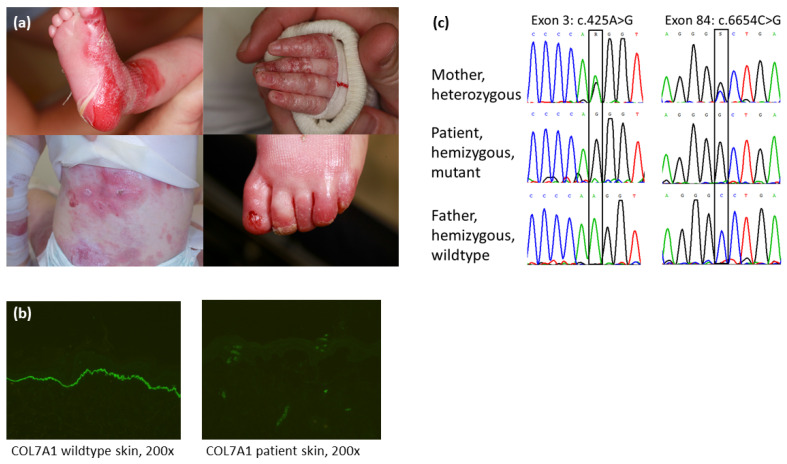
(**a**) Patient. Clinical features of severe RDEB with large areas of denuded skin (aplasia cutis) on the heels, partial toe fusion, and absence of toenails; (**b**) Immunofluorescence (IF) antigen mapping of the perilesional skin using antibody LH7.2 (Abcam, Cambridge, UK) staining against collagen type VII reveals dermal blistering and total absence of collagen type VII beneath the lamina densa compared to control; (**c**) Mutation analysis. Mutation c.425A>G as well as the single nucleotide polymorphism c.6654C>G are both hemizygous in the patient and confirmed by the heterozygous status of the mother. In contrast, both paternal loci show a wild-type profile.

Our index patient was delivered spontaneously as the second child of unrelated parents at the 39th week of gestation and after an otherwise uneventful pregnancy. At birth, she presented with large areas of denuded skin (Aplasia cutis) on both feet (particularly heels) as well as smaller erosions on her lips and oral mucosa. Within the first days of life, numerous blisters and erosions occurred with a predilection of mechanically exposed areas (Figure 1a), while her general condition (including weight, length, and head circumference), as well as vital parameters, were unsuspicious and within the normal range. Screening for the inflammatory, autoimmune, metabolic, infectious, or microbial causes of neonatal blistering using serology and microbial cultures determined no pathology. Immunofluorescence (antigen) mapping of the perilesional skin, performed in the first week of life, showed an absence of collagen type VII, consistent with the diagnosis of severe RDEB (Figure 1b). The mutation analysis of *COL7A1* finally confirmed the diagnosis (Figure 1c). Notably, a detailed exploration of the family history revealed that the daughter of the second cousin of the mother also suffered from *Epidermolysis bullosa* but was not confirmed by approaches of molecular biology. At the current age of 8 years, our patient presents with recurrent and extensive generalised cutaneous and mucosal blistering, chronic wounding, nail dystrophy, progressive contractures of the fingers and toes (pseudosyndactyly), as well as oesophageal strictures.

Revisiting the molecular pathology and applying advanced molecular diagnostics, this case is based on an exceptionally rare disease mechanism characterised by the hemizygosity for the hotspot mutation c.425A>G in the maternal *COL7A1* allele. This alteration harbours a high recurrence rate in DEB patients of central European origin, with an allelic frequency of 12.8–14%. Affecting exon 3, the molecular defect causes the anomalous transcriptional processing of pre-mRNAs [8,26,27]. It is accompanied by an extensive deletion on the second allele on chromosome 3p21.31, which almost completely erases the *COL7A1* gene and the adjacent flanking segments of downstream genes.

## 2. Materials and Methods

### 2.1. NGS-Based Mutation Analysis

Genomic DNA was isolated from the peripheral blood of the patient and his parents using an innuPrep Blood DNA mini kit (Analytik Jena, Jena, Deutschland). EB panel sequencing including the *COL7A1* gene and at least 16 genes involved in classical *Epidermolysis bullosa* [28] was performed on 10ng DNA of the patient with a Personal Genome Machine (PGM) of the Next Generation Sequencing Platform of Life Technologies according to the protocol of the company and analysed with the Ion Reporter Software (Life Technologies, Carlsbad, CA, USA). The mutation in exon 3 and the variant in exon 84 were amplified using PCR (Promega, Madison, GA, USA), confirmed via Sanger sequencing using a 3500 Series Genetic Analyzer (Applied Biosystems, Waltham, MA, USA), and compared with the reference sequence of *COL7A1* (NM_000094) using the BLAST algorithm.

### 2.2. Immunofluorescence (IF) Antigen Mapping

The samples were stained with primary mouse antibody LH7.2 diluted at 1:500 (Abcam, Cambridge, UK). A goat anti-mouse FITC conjugated secondary antibody was used in a dilution of 1:100 (Merck, Darmstadt, Germany, BRD).

### 2.3. PCR-Based STR Typing

Purified undiluted DNA from the reference samples (patient and her parents) was analysed utilising a Quantifiler Trio kit (Life Technologies, Carlsbad, CA, USA) [29] for DNA quantification. After normalising to 0.5 ng/µL, 1 and 2 µL of diluted DNA were amplified with an AmpFlSTR^®^NGMSElect^TM^ kit (Applied Biosystems, Waltham, MA, USA) [30] for STR/microsatellite analysis according to the manufacturers’ guidelines with a reduced reaction volume of 12.5 μL. Fragment length analysis was carried out on a 3500 Genetic Analyzer System (36 cm capillary array, POP4-polymer, Life Technologies, Carlsbad, CA, USA). Data analysis, allele calling, and genotyping employed the GeneMapper ID-X software (Applied Biosystems, Waltham, MA, USA) [31]. All lab work was performed according to the EN DIN ISO/IEC 17025:2005 guidelines [32]. To reassure the biological kinship between the patient and her parents, a forensic paternity test was carried out. Biostatistics was calculated utilising the “Program for biostatistical kinship analysis” [33] referring to allelic frequencies as seen in a western European reference population.

### 2.4. Copy Number Analysis via Multiplex Ligation-Dependent Probe Amplification (MLPA)

MLPA was performed to detect large heterozygous deletions of the *COL7A1* gene. The MLPA kit used in this study contains 29 probes for *COL7A1* (the gene responsible for dystrophic EB), 9 probes for KRT5 (the gene responsible for EBS), and 10 reference probes detecting different autosomal chromosome locations (SALSA MLPA Probe Mix P415-A1 *COL7A1*-*KRT5*; MRC-Holland, Amsterdam, The Netherlands).

DNA was denatured at 98 °C for 5 min, followed by the addition of the probe mixture, heated at 95 °C for 2 min, and incubated at 60 °C for 16 h. On day 2, ligase was added to the mixture, followed by incubation at 54 °C for 15 min. The mixture was then heated to 95 °C for 1 min to inactivate the ligase and was transferred to the PCR mix containing carboxyfluorescein-labelled primers. PCR was performed at 94 °C for 3 min, 30 cycles at 94 °C for 30 s, 60 °C for 45 s, and 68 °C for 15 s, and the reaction was ended with incubation at 68 °C for 7 min. The PCR products were then carried out on a 3500 Genetic Analyzer (Applied Biosystems, Waltham, MA, USA). The final analysis was performed with the CoffalyserNet Software 140701.0000 (MRC Holland, Amsterdam, The Netherlands).

### 2.5. Linear Amplification Mediated PCR (LAM-PCR)

**Figure 2:**
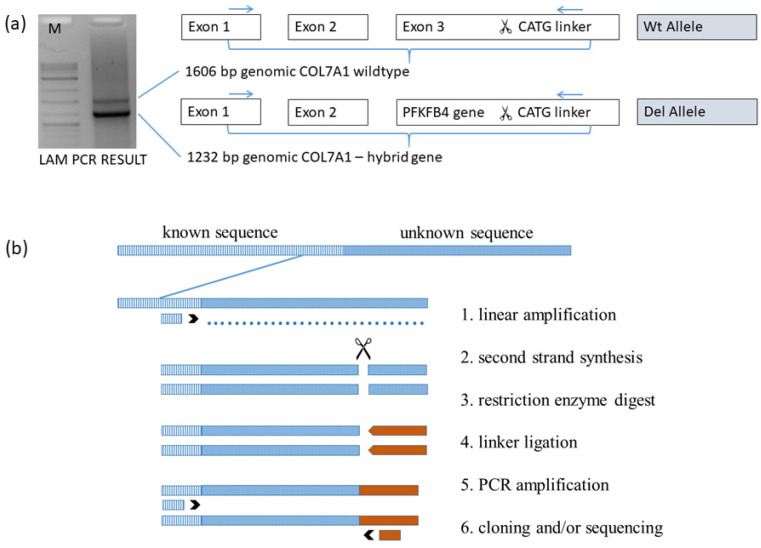
(**a**) Linear amplification mediated PCR (LAM-PCR) of the genomic *COL7A1* wild-type allele (including exon 1, intron 1, exon 2, intron 2, and part of exon 3), digested by NlaIII and conjugated with a CATG linker (upper band); genomic hybrid of *COL7A1* combining exon 1, intron 1, exon 2, part of intron 2 with section of *PFKFB4* gene, digested by NlaIII and conjugated with a CATG linker (lower band); (**b**) LAM-PCR overview. Different steps characterise this method starting with the forward primer (1. linear amplification) complementary to a known sequence (dashed), overriding the border to the unknown sequence (dotted). Producing the second strand (2. second strand synthesis) and cutting the sequence (scissors) by a 4-cutter enzyme (3. restriction enzyme digest) is followed by ligation of a linker cassette (brown bars) (4. linker ligation). The final chimeric product is generated/produced via PCR with a primer combination complementary to the known sequence and the linker sequence (5. PCR amplification). TA cloning of the PCR product into a plasmid and/or direct sequencing with Sanger is optional (6. Cloning and/or sequencing).

In this methodological approach to advance from a known DNA sequence to obtain an adjacent unknown sequence, a hybrid sequence was prepared for analysis via sequencing. The following description of this method is adapted according to the details described by Schmidt M et al. [34]. The first step is to obtain the linear amplified DNA strand, which was performed via PCR at 58 °C and 50 cycles using solely the forward primer biotin-*COL7A1*-1F3. The biotinylated linear PCR product was covalently linked to streptavidin-coupled magnetic beads overnight with a 300 rpm shaker. Each of the subsequent enzymatic steps was followed using a purification step of the beads via selection on a magnetic particle concentrator. After purification, the synthesis of the second strand was performed and continued with NlaIII restriction enzyme digest as well as the ligation of the NlaIII linker cassette (GTAC (+) GTAATACGACTCACTATAGGGCTCCGCTTAAGGGACCATG hybridised to GTAC (−) GTCCCTTAAGCGGAG). NaOH (100 µM) incubation for 15 min was used to release the second unbound DNA strand for application in the following PCR at 58 °C and 35 cycles using the primer biotin-*COL7A1*-1F2 combined with the linker primer LC1-rv. The obtained PCR product was again coupled to magnetic beads overnight using a 300 rpm shaker, followed by the detachment of the available DNA strand for application in the last PCR step at 58 °C and 35 cycles using the nested primer *COL7A1*-1F1 combined with the nested linker primer LC2-rv. The final PCR product was extracted and purified using agarose gel (Figure 2a) and the purifying kit, followed by optional TA cloning and/or sequencing with Sanger. A schematic following the different steps necessary in LAM-PCR is shown (Figure 2b).

Primer for LAM-PCR:

Biotin-*COL7A1*-1F3_ GGGACTTTTCTCTGCTCTGC

Biotin-*COL7A1*-1F2_ GGGAGCAAGGGACAGAGG

*COL7A1*-1F1_CGGCTTTTACTGCCTAGGAT

LC1rv_ GTAATACGACTCACTATAGGGC

LC2rv_ AGGGCTCCGCTTAAGGGAC

### 2.6. Confirmation of the 40 kb Deletion with PCR

To confirm the deletion with PCR, a primer pair was constructed (*COL7A1*-Intr2F1_AGTGCAGTACAGCGATGACC; *PFKFB4*-Intr1R1_GCAACTGCTCTCACCTCCTT) which was located at 5′prime and 3′prime of the deletion, in the *COL7A1* and the *PFKFB4* gene, respectively.

## 3. Results

### 3.1. Immunofluorescence (IF) Antigen Mapping

The IF of the patient’s perilesional skin biopsy, performed in the first week of life, determined dermal blistering and a total absence of collagen type VII beneath the lamina densa compared to the wild-type control skin. Antibody LH7.2 (Abcam, Cambridge, UK) was used to stain against collagen type VII. This result was consistent with the diagnosis of severe recessive dystrophic *Epidermolysis bullosa* (RDEB) (Figure 1b).

### 3.2. Genotyping Discrepancy of c.425A>G and c.6654C>G within the Family

The Sanger sequencing of the patient revealed a single chromatographic peak for the mutation c.425A>G in exon 3 and the variant c.6654C>G in exon 84 of *COL7A1*. The proband’s mother showed a heterozygous status for the mutation and the variant, whereas the paternal samples indicated a wild-type sequence for both segments (Figure 1c).

### 3.3. Paternity Testing

All the alleles detected by fragment length analysis revealed at least one matching allele from the father and mother in the patient’s genotype (Table 1, upper part). The “Program for biostatistical kinship analysis” [33] resulted in a probability of 99.99999999998% for the hypothesis that the patient indeed is the daughter of the reputed parents.

### 3.4. STR Marker Test on Chromosome 3 Adjacent COL7A1

Two microsatellite markers (D3S1289, D3S3629) upstream *COL7A1* and one microsatellite marker (D3S1581) downstream were analysed via fragment length analysis running on a 3500 Genetic Analyzer System, followed by data analysis, allele calling, and genotyping using the GeneMapper software. Marker D3S3629 in the vicinity upstream of *COL7A1* was determined to be heterozygous, whereas marker D3S1581 near downstream of *COL7A1* was homozygous. (Table 1, lower part).

These results suggested either short segmental maternal isodisomy leading to homozygosity or the deletion of the paternal allele resulting in hemizygosity, both mainly including the regions downstream *COL7A1* as a possible mechanism for the genotyping discrepancies.

### 3.5. Copy Number Analysis Revealed COL7A1 Hemizygosity of the Patient

**Figure 3:**
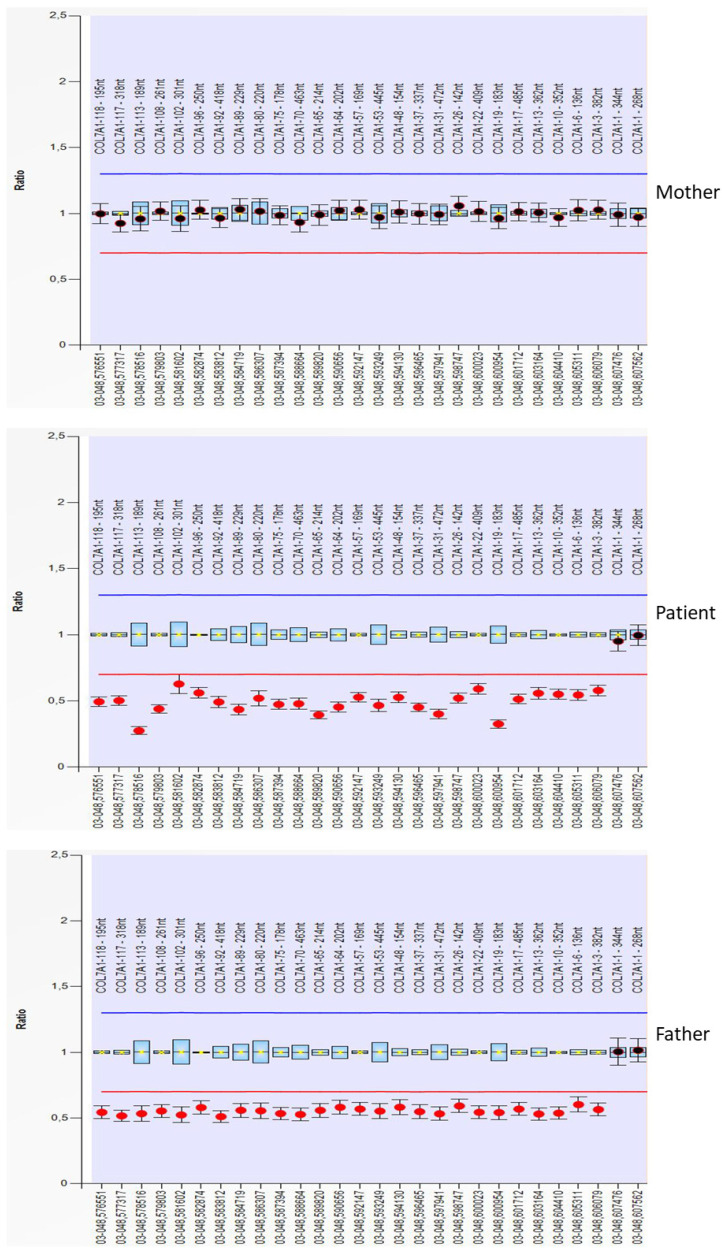
Multiplex ligation-dependent probe amplification (MLPA) copy number analysis of the patient and his parents revealed hemizygosity ranging from exon 3 to 118 in the patient and his father. A set of probes is located and distributed on the exons of *COL7A1*. Exon 1 is covered by two different probes. Exon 2 is not covered.

To test if isodisomy or hemizygosity is predominating, we performed multiplex ligation-dependent probe amplification (MLPA) examining the *COL7A1* copy number in the patient and her parents. The patient and her father were found to be hemizygous for most of the *COL7A1* exons tested (27 probes for 27 different exons of *COL7A1*), except 2 probes for exon 1, while her mother displayed a normal *COL7A1* copy number (Figure 3). All the tested family members were normal for 19 control exons located outside of chromosome 3 (9 probes for KRT5 and 10 reference probes, not shown). Collectively, our findings favoured a familial deletion of the paternal allele, resulting in the hemizygosity of part of the chr3p21.31 region spanning *COL7A1*, rather than indicating UPD, to underlie this RDEB case.

### 3.6. Linear Amplification Mediated PCR Exactly Defined the Breaking Points of Deletion

**Figure 4:**
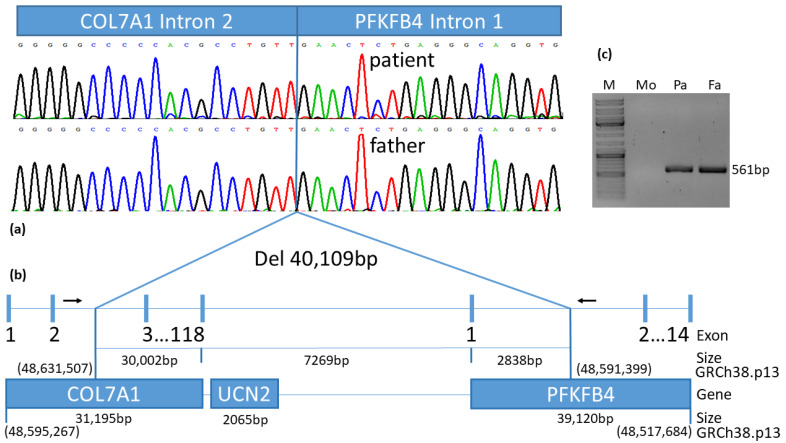
Schematic illustration of the ~40kb deletion: (**a**) the hybrid sequence of *COL7A1* intron 2 combined with *PFKFB4* intron 1 is shown for patient and his father; (**b**) confirming a deletion of 40,109 bp covering a large part of *COL7A1* that includes exons 3 to 118, the complete *UCN2* gene and its neighbouring intragenic region as well a small part of *PFKFB4* gene comprising exon 1 and a section of intron 1. Primer binding sites for deletion confirmation are drawn as black arrows. Bold numbers delineate the exons of the genes. Size of defined regions is stated as the number of base pairs (bp). Genomic positions are denoted by GRCh38.p13; (**c**) PCR amplification of the 561 bp fragment, spanning the 40 kb deletion, confirms the deletion for the patient and his father, as shown on agarose gel.

The final products of LAM-PCR amplified for the patient and his father resembled a hybrid sequence of *COL7A1* intron 2 at the genomic position (chr3:48,631,507), which is linked to *PFKFB4* intron 1 at the genomic position (chr3:48,591,399) (Figure 4a,b). An extensive deletion comprises 40,109 bp including *COL7A1* exon 3 to 118 (30,002 bp), proximate *UCN2* gene with the neighbouring intragenic region (7269 bp), and the upstream region of *PFKFB4* gene with exon 1 and part of intron 1 (2838 bp).

### 3.7. Confirmation of the 40 kb Deletion via PCR

PCR was performed with a primer pair located distal and proximal to the deletion, showing a calculated fragment size of 561 bp in case of an existing deletion. The agarose gel depicted a distinct band for the patient and the father, confirming the deletion. In contrast, the mother reflected a wild-type status with the absence of the band (Figure 4c).

## 4. Discussion

Our results highlight the necessity to consider large *COL7A1* deletions with subsequent hemizygosity of the second allele as well as the occurrence of UPD in cases where a homozygous mutation is being detected in a DNA sequence chromatogram.

In this context, the delineation of pathogenic sequences may be challenging. A DNA sequence chromatogram is incapable to distinguish the distinct, exceptionally rare molecular events of UPD and deletion-based hemizygosity. Moreover, as a hemizygous mutation may mimic a homozygous molecular aberration, it is essential to perform mutation analysis not only in the index patient but also in both parents whose biological identity can be confirmed by paternity testing. Consistently, a comparison of patient and parental Sanger sequences may point to a genotyping discrepancy, as seen in our case (Figure 1c).

While the testing of STR markers on chromosome 3 adjacent to *COL7A1* confirmed heterozygosity upstream to D3S3629, the downstream homozygosity shown by D3S1581 indicated genotyping discrepancies (Table 1, lower panel). Possible mechanisms included either homozygosity due to a rather unusual paternal short segmental maternal isodisomy or alternatively hemizygosity caused by allele deletion. Both traits would focus on *COL7A1* and downstream regions.

Multiplex ligation-dependent probe amplification for *COL7A1* was used to determine the molecular basis in this study. Thereby, hemizygosity was detected for most probes of *COL7A1* tested in the patient and her father, favouring a familial gross deletion of the paternal *COL7A1* allele in the chr3p21.31 region.

An important consequence of hemizygosity is that any residual collagen type VII function is strictly dependent on one remaining allele. Referring to the literature, the first intragenic large deletion in *COL7A1* was published by Kern et al. [23]. The breakpoints were found to span over 4kb starting in intron 12 and ending in exon 24. The homozygous deletion was designated as c.1637−240_3255del4064. The parents were shown to be heterozygous. A rather short heterozygous deletion affecting exons 1 and 2 in COL7A1 was reported by Aradhya et al. [22]. Designated as chr3:48,606,924-48,607,775, the mutation led to an 851 bp deletion in combination with the insertion c.3942dupG.

The exact definition of both breakpoints involved in the deletion within and proximal to the *COL7A1* gene may be challenging. Although genome sequencing may accurately identify these areas, it is a material, time, and cost-consuming option. Exome sequencing allows only the detection of deleted exons, and fine-tuning in intronic regions is largely limited.

Considering the fact that the distal breakpoint could be restricted to the downstream region of COL7A1 exon 2, as estimated by MLPA, we applied LAM-PCR using a designed linear primer located in exon 1. Thereby, both breakpoints were targeted within intron 2 (chr3:48,631,507) of the *COL7A1* gene, involving a 40kb sequence within intron 1 (chr3:48,591,399) of the adjacent *PFKFB4* gene.

The LAM-PCR data further showed that, in addition to *COL7A1*, the patient and his father were hemizygous for two other genes (*UCN2* total and *PFKFB4* partial) (Figure 4; Table 2). While the patient displays prototypic features of RDEB, there is currently no hint of a phenotypic impact of these genes in our patient. This is consistent with cases reported in the literature [24,25]. Thus, Lee et al. [24] reported a de novo deletion of the maternal allele, resulting in hemizygosity of a 368kb region of chromosome 3 including *COL7A1* and another 15 contiguous genes. The second-site paternal splice mutation in intron 53 led to the exon skipping of exon 53 responsible for the blistering skin condition. Titeux et al. [25] described a paternally inherited deletion and provided an estimate of the minimum span of the deletion at 255kb and a maximum potential span of 520kb encompassing 9 to 15 genes. The maternal hemizygous mutation in exon 94 was involved in aberrant splicing and responsible for the manifestation of RDEB. In the latter both hemizygous cases, the exact breaking points were not estimated due to methodologic restrictions.

Apart from *COL7A1*, none of the genes within the deletion interval of our patient has hitherto been associated with a distinct dominant or recessive disorder. According to the DECIPHER database (https://www.deciphergenomics.org/search) (accessed on 1 August 2021), the haploinsufficiency indices of *PFKFB4* (%HI = 51.14) and *UCN2* (%HI = 77.92) indicate that these genes are more likely to not exhibit haploinsufficiency [35]. However, monitoring for late-onset tumorigenesis is indicated, as *PFKFB4* expression is involved in glycolysis and regulated by several molecular pathways, including those closely linked to oncogenic signalling [36]. Consistently, *PFKFB4* is overexpressed in numerous malignancies, and its knockdown (with a molecular status comparable to haploinsufficiency) was demonstrated to inhibit proliferation and invasiveness in IHH-4 thyroid cancer cells alongside the upregulation of histone acetyltransferase GCN5 [37].

### Genetic Counselling

**Figure 5:**
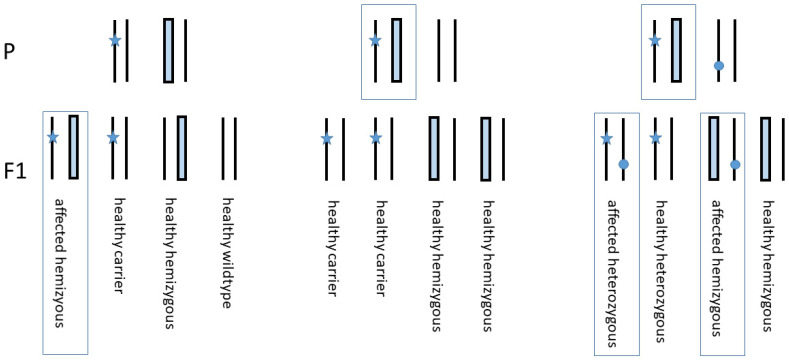
Genetic segregation for genetic counselling. Left panel shows the segregation of the index/affected family with the mother as a carrier and the hemizygous father. Likelihood of recurrence of an affected child is 25%, and a healthy child is expected at 75% (carrier 25%, hemizygous wild-type 25%, wild-type 25%). The central panel identifies all healthy siblings of the affected patient in case of a wild-type partner (50% carrier of the mutation, 50% hemizygosity wild-type due to deletion). If the partner of an affected patient is a heterozygous carrier of a pathogenic mutation (right panel), 50% of their offspring will be affected (25% combined heterozygous, 25% hemizygous mutant), and 50% will be healthy (25% carrier, 25% hemizygous wild-type). Star, maternal mutation; filled rectangle, deleted hemizygous paternal allele; circle, suspected pathogenic mutation; affected person marked by rectangle; P, parental generation, F1, offspring generation.

Regarding genetic counselling, the parents of a hemizygous child are informed that the recurrence of an affected child is 25%, while the probability of a healthy carrier is 75% (carrier of mutation 25%, carrier of hemizygous wild-type allele 25%, and wild-type 25%) (Figure 5 (left panel)). A clinically unaffected sibling of the index patient is expected to carry either the mutation in 50% or is hemizygous wild-type in 50% (middle panel).

However, the *COL7A1* gene of the partner of an affected individual should be predictively analysed to determine a heterozygous carrier status, which would account for a 25% occurrence of a hemizygous mutant phenotype in any other sibling as well as the 25% probability of combined heterozygosity (right panel). Against this background, prenatal diagnosis is of considerable value in the context of severe RDEB.

## 5. Conclusions

This report describes an RDEB patient with a hemizygous 40kb deletion of *COL7A1* and adjacent genes. In addition to a single nucleotide polymorphism in exon 84, the non-deleted (maternal) allele revealed a second-site base substitution in the 3′-end of exon 3. The latter aberration, c.425A>G, is well-known as a hotspot mutation that induces a pre-mRNA splicing defect in combination with the amino acid change (p.K142R) [27]. Our findings underscore the importance of a “holistic” approach to mutational profiling to also characterise exceptionally rare pathogenic sequences such as incongruent gene transmission as a prerequisite for accurate genetic counselling in RDEB. Linking this methodology with other techniques at the epigenetic and protein level [38,39] will foster the identification of pathogenic mediators and therapeutic targets to combat this devastating disease.

## Figures and Tables

**Table 1:** Haplotype analysis of the family. The upper part of the table depicts the patient’s and her parent’s alleles according to the AmpFlSTR^®^NGMSElect^TM^-kit loci. Matching alleles are highlighted in colour (patient and mother in brown, patient and father in blue). Selected loci surrounding the *COL7A1* gene, including the mutation in exon 3 and the variant in exon 84, are shown in the lower part of the table.

MS Marker	Mother		Patient		Father	
D10S1248	14	15	14	14	14	14
vWA	17	16	17	15	15	15
D16S539	11	12	11	9	14	9
D2S1338	25	18	25	26	17	26
AMEL	X	X	X	Y	X	Y
D8S1179	14	12	14	13	11	13
D21S11	30	29	30	29	29	29
D18S51	16	13	16	18	21	18
D22S1045	16	15	16	11	15	11
D19S433	13	14	13	14	15	14
TH01	8	7	8	9.3	9	9.3
FGA	22	20.2	22	22	24	22
D2S441	14	14	14	14	14	14
D3S1358	18	14	18	15	15	15
D1S1656	15	16.3	15	18.3	16	18.3
D12S391	18	18	18	16	17.3	16
SE33	14	19	14	14	29.2	14
D3S1581	86	111	86	86	84	86
* *COL7A1* c.6654C>G (ex84)	**var**	**wt**	**var**	**del**	**wt**	**del**
* *COL7A1* c.425A>G (ex3)	**mut**	**wt**	**mut**	**del**	**wt**	**del**
D3S3629	246	244	246	250	250	250
D3S1289	200	189	200	200	202	200

* Defines the locus of the mutation in exon 3 and the variant in exon 84 (rows in bold); mut/var defines the occurrence of the mutation/variant; wt, wild-type; del, deletion.

**Table 2:** Genes involved in hemizygous 3p21.31 deletion.

Gene Symbol ^1^	Gene Name ^1^	Protein Function	OMIM ^2^
*COL7A1*	Collagen type VII, alpha 1 (*Epidermolysis bullosa*, dystrophic, dominant and recessive)	Assembles into anchoring fibrils, ensuring adherence of epidermis to the underlying dermis.	120120
*UCN2*	Urocortin 2	Specific ligand for the type 2 CRH receptor, which mediates stress coping responses during the recovery phase of stress.	605902
*PFKFB4*	6-phosphofructo-2-kinase/fructose- -2,6-biphosphatase 4	Regulates the steady-state concentration of fructose-2,6-bisphosphate in the glycolysis pathway.	605320

^1^ HUGO-Gene Nomenclature Committee approved gene symbols and names were used; ^2^ Online Mendelian Inheritance in Man: http://www.ncbi.nlm.nih.gov/omim/ (accessed on 1 August 2021)

## Data Availability

Authors confirm that the data supporting the findings of this study are available within the article.

## Abbreviations

GRCh38.p13.	Genome Reference Consortium Human Build 38 patch release 13
*COL7A1*	Collagen type VII, alpha 1
*PFKFB4*	6-Phosphofructo-2-kinase/fructose2,6-biphosphatase 4
*UCN2*	Urocortin 2
UPD	Uniparental isodisomy
LAM-PCR	Linear amplification-mediated PCR
MLPA	Multiplex ligation-dependent probe amplification
